# Stress Modifies the Expression of Glucocorticoid-Responsive Genes by Acting at Epigenetic Levels in the Rat Prefrontal Cortex: Modulatory Activity of Lurasidone

**DOI:** 10.3390/ijms22126197

**Published:** 2021-06-08

**Authors:** Paola Brivio, Giulia Sbrini, Letizia Tarantini, Chiara Parravicini, Piotr Gruca, Magdalena Lason, Ewa Litwa, Chiara Favero, Marco Andrea Riva, Ivano Eberini, Mariusz Papp, Valentina Bollati, Francesca Calabrese

**Affiliations:** 1 Department of Pharmacological and Biomolecular Sciences, Università degli Studi di Milano, 20133 Milan, Italy; paola.brivio@unimi.it (P.B.); giulia.sbrini@unimi.it (G.S.); chiara.parravicini@unimi.it (C.P.); m.riva@unimi.it (M.A.R.); ivano.eberini@unimi.it (I.E.); 2 EPIGET Lab, Department of Clinical Sciences and Community Health, Università degli Studi di Milano, 20122 Milan, Italy; letizia.tarantini@unimi.it (L.T.); chiara.favero@unimi.it (C.F.); valentina.bollati@unimi.it (V.B.); 3 Maj Institute of Pharmacology Polish Academy of Sciences, 31-343 Krakow, Poland; gruca@if-pan.krakow.pl (P.G.); mlason@if-pan.krakow.pl (M.L.); litwa@if-pan.krakow.pl (E.L.); nfpapp@cyfronet.pl (M.P.)

**Keywords:** stress, epigenetic, DNA methylation, miRNA, GR-responsive genes, lurasidone

## Abstract

Epigenetics is one of the mechanisms by which environmental factors can alter brain function and may contribute to central nervous system disorders. Alterations of DNA methylation and miRNA expression can induce long-lasting changes in neurobiological processes. Hence, we investigated the effect of chronic stress, by employing the chronic mild stress (CMS) and the chronic restraint stress protocol, in adult male rats, on the glucocorticoid receptor (GR) function. We focused on DNA methylation specifically in the proximity of the glucocorticoid responsive element (GRE) of the GR responsive genes Gadd45*β*, Sgk1, and Gilz and on selected miRNA targeting these genes. Moreover, we assessed the role of the antipsychotic lurasidone in modulating these alterations. Chronic stress downregulated *Gadd45β* and *Gilz* gene expression and lurasidone normalized the *Gadd45β* modification. At the epigenetic level, CMS induced hypermethylation of the GRE of *Gadd*45*β* gene, an effect prevented by lurasidone treatment. These stress-induced alterations were still present even after a period of rest from stress, indicating the enduring nature of such changes. However, the contribution of miRNA to the alterations in gene expression was moderate in our experimental conditions. Our results demonstrated that chronic stress mainly affects Gadd45β expression and methylation, effects that are prolonged over time, suggesting that stress leads to changes in DNA methylation that last also after the cessation of stress procedure, and that lurasidone is a modifier of such mechanisms.

## 1. Introduction

Brain disorders have a multifactorial etiology and several genetic and environmental factors have been recognized to play a key role in the development of these pathologies. In this context, stressful life events are among the environmental factors that have been strictly associated with the onset of major depressive disorders [1,2].

Among the systems activated in response to exposure to negative stimuli, the hypothalamic-pituitary adrenal (HPA) axis is the primary system involved in stress response and it has also been demonstrated that patients affected by mood disorders are characterized by alteration in the HPA activity [3,4,5].

Accordingly, we recently showed that, at preclinical levels, exposure to chronic stress induced depressive-like behavior in adult male rats and affected the HPA functions, by altering both the genomic and non-genomic mechanisms of the glucocorticoid receptors (GR) [6]. In detail, the GR may regulate different function depending on its subcellular localization, with the cytosolic GR that translocate in the nucleus after the binding with glucocorticoids mediating the transcription of gene presenting the glucocorticoid responsive element (GRE) (genomic pathway) and the GR located on the neuronal and mitochondrial membranes regulating the release of neurotransmitters and/or the activity of the mitochondria (non-genomic signaling) [3].

Further, we observed that chronic treatment with the novel multimodal antipsychotic lurasidone, receptor antagonist D2, 5-HT2, and 5-HT7 (LUR) was able to normalize the behavioral and the molecular alterations induced by prolonged stress exposure [6]. In particular, stress inhibited the transcription of genes containing the GRE following a cognitive task and chronic LUR treatment restored these alterations in stressed animals [6].

Here, among the regulatory mechanisms of gene expression, we investigated whether transcriptional changes caused by chronic stress exposure may be sustained by epigenetic modifications, in particular by alteration in DNA methylation and miRNA expression, mechanisms known to be modulated by environmental factors as well as by drug treatments [7,8,9,10,11,12]. Indeed, the epigenetic make-up may be persistently changed in response to social and physical factors, including adverse stimuli, thus affecting brain functions and neurobiological processes [9,13,14,15,16,17,18,19,20,21,22], and in human, several reports indicated changes in DNA methylation in the blood of depressed patients [23,24,25].

On these bases, this study aims to investigate the potential contribution of epigenetic mechanisms, such as DNA methylation and miRNA expression, in the alteration of the transcription of genes regulated by GR activation, in two animal models useful for the study of depression: the chronic mild stress and the chronic restraint stress protocols. In particular, we measured the methylation status of the cytosine present in the proximity of the GRE sequences of selected genes named *Gadd45β*, *Sgk1,* and *Gilz* to more deeply investigate the activity of GR as a transcription factor and the accessibility to its responsive element on the DNA.

These genes have been widely associated with GR action, HPA functioning, and psychopathologies, [26,27,28] and their expression is mediated by the binding of nuclear GR with the GRE [28,29,30,31,32].

We then assessed the influence of the pharmacological intervention with LUR in modulating the transcription of *Gadd45β*, *Sgk1,* and *Gilz* possibly by acting at the epigenetic level. Finally, by measuring the methylation status of these genes and the potential involvement of miRNA in the stress effects following a period of rest, we explored whether the effects exerted by stress exposure were long-lasting.

The analyses were performed in brain regions strictly connected with stress, such as the prefrontal cortex (PFC), the ventral (vHip), and the dorsal (dHip) hippocampus.

## 2. Results

### 2.1. Effect of Chronic Mild Stress and Lurasidone Treatment on Body Weight and Sucrose Intake Measured in the Sucrose Consumption Test

Chronic stress led to a significant reduction of body weight, as indicated by the two-way ANOVA analysis (F_1–39_: 5.396, *p* = 0.026). Seven weeks are needed to obtain this effect (−8%, *p* < 0.05 vs. No stress) that is not observed after 3 weeks of exposure (Table S1a). On the contrary, the pharmacological treatment did not affect the body weight both in control and in stressed rats, neither after 3 weeks of CMS nor at the end of the CMS procedure.

Moreover, in line with our previous results [6,33,34], after 3 weeks of CMS, we observed a significant reduction in sucrose intake (−50%, *p* < 0.01 vs. No stress/VEH, Tukey multiple comparison’s test) in comparison to control animals as revealed by the significant effect of stress (F_1–39_: 20.1, *p* = 0.000) in the two-way ANOVA analysis (Table S1b). Furthermore, the anhedonic-like behavior was also present after 7 weeks of CMS. Indeed, a two-way ANOVA analysis revealed a significant effect of stress (F_1–39_: 8.499, *p* = 0.0061) and a significant stressXtreatment interaction (F_1–39_: 7.130, *p* = 0.0113), with the sucrose intake being reduced at week 7 of CMS (−52%, *p* < 0.01 vs. No stress/VEH, Tukey multiple comparison’s test), an effect that was completely normalized by prolonged lurasidone treatment (+74%, *p* < 0.05 vs. CMS/VEH, Tukey multiple comparison’s test). Similar effects were found in the two-way ANOVA when correcting the sucrose intake by the weight of the rats (Table S1c), with a significant effect of stress (3 weeks: F_1–39_: 16.934, *p* = 0.000; 7 weeks: F_1–39_: 5.706, *p* = 0.022) and a significant stressXtreatment interaction after 7 weeks of CMS (F_1–39_: 5.836, *p* = 0.021).

### 2.2. Modulation of Gadd45β, Sgk1, and Gilz Gene Expression of Rats Exposed to the CMS and Treated with Lurasidone

To establish the effect of chronic stress and lurasidone treatment on GR-downstream effectors, we analyzed the mRNA levels of three GR responsive genes, namely *Gadd45β*, *Sgk1,* and *Gilz*.

**Figure 1:**
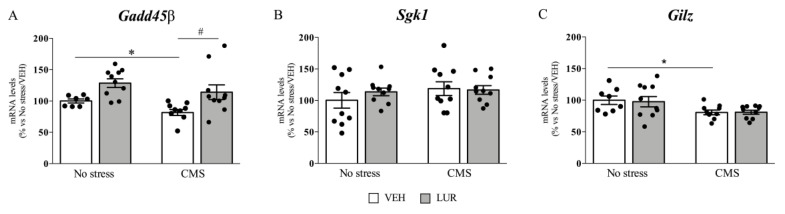
Analysis of *Gadd45β* (**A**) *Sgk1* (**B**) and *Gilz* (**C**) mRNA levels in the prefrontal cortex of chronically stressed rats: Modulation by chronic lurasidone (LUR) treatment. The data, expressed as a percentage of No stress/VEH animals (set at 100%), are the mean ± SEM. * *p* < 0.05 vs. No stress/VEH; # *p* < 0.05 vs. CMS/VEH (Two-way ANOVA with Tukey multiple comparison’s test).

As shown in Figure 1A, we found a significant effect of stress (F_1–36_: 4.840, *p* = 0.035, two-way ANOVA) as well as of the treatment (F_1–36_: 6.971, *p* = 0.013, two-way ANOVA) on *Gadd45β* expression in rat PFC. Indeed, 7 weeks of CMS produced a significant reduction of *Gadd45β* mRNA levels (−18%, *p* < 0.05 vs. no stress/VEH, Tukey multiple comparison’s test) compared to non-stressed rats. On the contrary, prolonged LUR treatment was able to normalize the CMS-induced downregulation of *Gadd45β* (+40%, *p* < 0.05 vs. CMS/VEH, Tukey multiple comparison’s test). By contrast, in both vHip (Table S2a) and dHip (see [6]), *Gadd45β* was not affected by stress or by the pharmacological treatment.

In line with what observed for *Gadd45β* in PFC the expression of *Gilz* was significantly modulated by stress (F_1–36_: 9.293, *p* = 0.005, two-way ANOVA), with *Gilz* mRNA levels being downregulated by chronic stress exposure (−19%, *p* < 0.05 vs. no stress/VEH, Tukey multiple comparison’s test) (Figure 1C), whereas we did not observe any modulation of its expression in vHip (Table S2a) and dHip (Table S2b).

Conversely, neither stress exposure nor pharmacological treatment modulated *Sgk1* gene expression in the PFC (Figure 1B) and dHip [6]. By contrast, in vHip, two-way ANOVA analysis revealed a significant effect of the treatment (F_1–39_: 17.290, *p* = 0.000). Indeed, *Sgk1* gene expression was decreased by LUR in CMS animals (−27%, *p* < 0.01 vs. no stress/VEH, Tukey multiple comparison’s test) (Table S2a).

### 2.3. Modulation of Gadd45β and Sgk1 DNA Methylation Levels in the PFC of Rats Exposed to the CMS and Treated with Lurasidone

**Figure 2:**
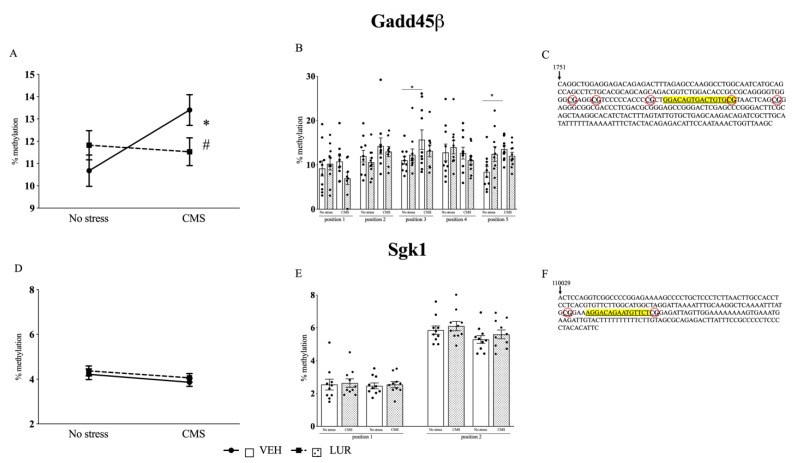
Analysis of Gadd45β (**A**,**B**) and Sgk1 (**D**,**E**) DNA methylation in the prefrontal cortex of chronically stressed rats (CMS): Modulation by chronic lurasidone (LUR) treatment. Panel (**C**–**F**): Schematic representation of transcriptionally relevant CpG sites (surrounded in red) in Gadd45β (**C**) and Sgk1 (**F**) in the vicinity of GRE (highlighted in yellow). The data, expressed as methylation percentage, are the mean ± SEM of the positions analyzed. * *p* < 0.05 No stress/VEH; # *p* < 0.05 vs. CMS/VEH (Two-way ANOVA with Tukey multiple comparison’s test).

Next, based on literature data [29], on the DNA sequences of the GR responsive genes we identified the possible GRE and we constructed the assay in these specific sub-regions, thus measuring the methylation status of the CGs here in. Interestingly, on Gadd45β we investigated the methylation of 5 CGs on, or nearby, the GRE (Figure 2C), and 2 CGs on Sgk1 GRE (Figure 2F), whereas in the proximity of the GRE of Gilz, there are no CGs.

Since we found the main effect of stress and treatment on gene transcription in the PFC, we explored the potential contribution of epigenetic mechanisms specifically in this brain region.

For Gadd45β, we found that chronic stress exposure induced hypermethylation of the CGs sited in the GRE (13.39% ± 0.7, *p* < 0.05 vs. no stress/VEH, Tukey multiple comparison’s test) as compared to non-stressed group (10.70% ± 0.7) (Figure 2A), an effect that is in agreement with the reduction observed for *Gadd45β* mRNA levels expression in the same group, as described above (Figure 1A). Interestingly, LUR treatment produced a statistically significant decrease of the methylation percentage in chronically stressed rats (11.31% ± 1, *p* < 0.05 vs. CMS/VEH, Tukey multiple comparison’s test) (Figure 2A), an effect that may contribute to the normalization of *Gadd45β* mRNA levels observed in CMS rats treated with LUR (Figure 1A). Based on the analysis of the different CGs, the effect of CMS was due to the methylation changes of the CG in position 3 (15.7% ± 2.2, *p* < 0.05 vs. no stress/VEH, Tukey multiple comparison’s test) and in position 5 (13.6% ± 0.8, *p* < 0.05 vs. no stress/VEH, Tukey multiple comparison’s test) (Figure 2B).

We also analyzed the methylation status of the two CGs present in the GRE sequence on Sgk1 DNA. In line with the mRNA expression, we did not find any changes due to stress procedure or pharmacological treatment, neither for the mean (Figure 2D) nor for the single position considered (Figure 2E).

### 2.4. Analysis of Gadd45β Sgk1, and Gilz Gene Expression in the Prefrontal Cortex of Chronically Stressed (CRS) Rats after a Period of Washout

Next, we decided to investigate whether the GR function could be altered by CRS exposure following a washout from the adverse experience. Indeed, while it is known that HPA axis activity is compromised by chronic stress exposure, little is known about the persistent consequences of prolonged negative adversities on its function.

Hence, we investigated the mRNA levels of *Gadd45β Sgk1,* and *Gilz* in stressed rats exposed to the CRS after 3 weeks of washout from stress exposure.

**Figure 3:**
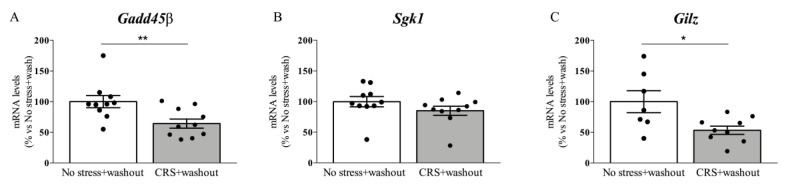
Analysis of *Gadd45β* (**A**) *Sgk1* (**B**) and *Gilz* (**C**) mRNA levels in the prefrontal cortex of chronically restraint stressed rats (CRS) after 3 weeks of washout. The data, expressed as a percentage of no-stress animals (set at 100%), are the mean ± SEM. * *p* < 0.05, ** *p* < 0.01 vs. No stress (Unpaired *t*-test).

As shown in Figure 3A, we found a significant decrease of *Gadd45β* mRNA levels (−36%, *p* < 0.01 vs. No stress; T value = 2.897) in chronically stressed rats following 3 weeks of washout. Similarly, *Gilz* expression was also significantly downregulated by CRS after the rest period (−47%, *p* < 0.05 vs. No stress; T value = 2.669) (Figure 3C), whereas a slight, but not significant, reduction was observed for *Sgk1* mRNA levels (−15%, *p* > 0.05 vs. No stress; T value = 1.327) (Figure 3B).

### 2.5. Analysis of Gadd45β and Sgk1 DNA Methylation Levels in Chronically Stressed (CRS) Rats after a Period of Washout

To evaluate whether the long-lasting effects of chronic stress exposure were sustained by epigenetic mechanisms, we investigated the DNA methylation levels in the same positions described above (Figure 2) of Gadd45β and Sgk1 after three weeks of rest from the stress procedure (CRS).

**Figure 4:**
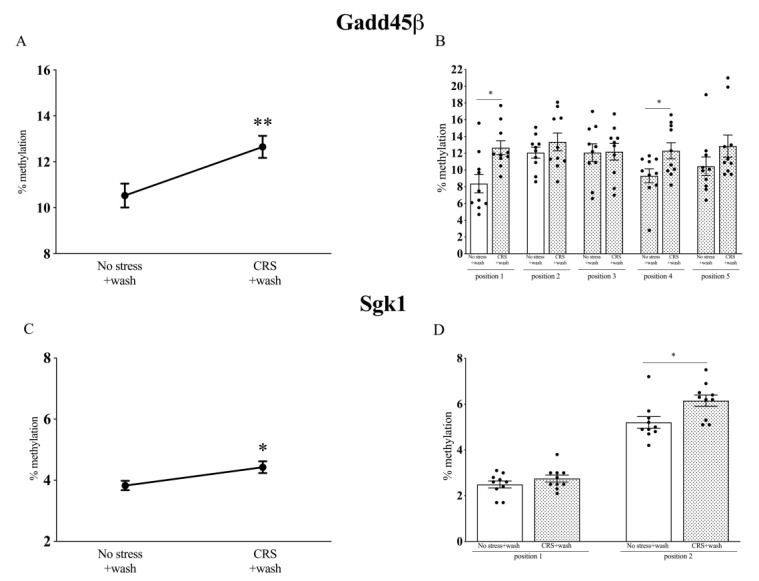
Analysis of Gadd45β (**A**,**B**) and Sgk1 (**C**,**D**) DNA methylation in the prefrontal cortex of chronically stressed rats (CRS) after 3 weeks of washout. The data, expressed as methylation percentage, are the mean ± SEM of the positions analyzed. * *p* < 0.05, ** *p* < 0.01 vs. No stress (Unpaired *t*-test).

As shown in Figure 4A, Gadd45β was significantly more methylated after three weeks of stress washout (12.64 ± 0.5, *p* < 0.05 vs. No stress; T value = 2.995) as compared to non-stressed rats (10.54 ± 0.5), in line with the decrease of its mRNA levels (Figure 3A), suggesting that this epigenetic mechanism may contribute to the persistent changes observed for this GR responsive gene. In particular, we found a specific increase in the methylation levels of the CGs in positions 1 (12.7 ± 0.8 vs. No stress; T value = 3.121) and 4 (12.3 ± 1.0 vs. No stress; T value = 2.348) (Figure 4B).

Similarly, Sgk1 methylation levels were significantly increased after the washout period from CRS (4.4 ± 0.2 vs. No stress; T value = 2.472), as compared to the no-stress group (3.8 ± 0.2) (Figure 4C), with a major contribution of the methylation of the position 2 (6.1 ± 0.3 vs. No stress; T value = 2.664) (Figure 4D), even if the *Sgk1* mRNA levels were not significantly altered in our experimental conditions (Figure 3B).

### 2.6. Effect of Stress and Lurasidone Treatment on miRNAs Expression

Lastly, we focused on another mechanism responsible for the control of gene expression, the miRNAs, that are emerging as a major regulator of psychiatric diseases [35,36,37].

Four miRNA families able to recognize the investigated mRNAs were identified: rno-mir-452-3p for Gadd45β, rno-miR-19b-3p and rno-miR-19a-3p for Sgk-1, and rno-miR-143-3p for Gilz (tsc22d3). These miRNAs were evaluated in our experimental setup. In the countercheck procedure, both *Gadd45β* and *Sgk-1* mRNAs were confirmed using both MicroT-CDS and miRDB, whereas the *Gilz* (tsc22d3) mRNA was not retrieved by the selected search tools.

The expression profile of miRNAs is presented in Table 1a,b. We found that miR-19a-3p was upregulated by chronic lurasidone treatment independently from pre-exposure to CMS (No stress: +78%, *p* < 0.05 vs. No stress/VEH; CMS: +100%, *p* < 0.01 vs. CMS/VEH, Tukey multiple comparison’s test), as indicated by the two-way ANOVA (F_1–34_: 15.800, *p* = 0.000). miR-143-3p was slightly reduced by chronic stress exposure (−39%, *p* = 0.064) and this alteration was partially normalized by lurasidone treatment (+55%, *p* = 0.067).

On the contrary, neither miR-452-3p nor miR-19b-3p were modulated neither by 7 weeks of chronic mild stress nor by the prolonged pharmacological administration.

Finally, we investigated whether the expression of these miRNAs may be altered after the period of washout from CRS. Interestingly, we observed that miR-143-3p was still reduced after three weeks of washout (−37%, *p* < 0.05 vs. No stress; T value = 2.306), whereas the other miRNAs considered were not significantly affected (Table 1b).

## 3. Discussion

In this study, we provide evidence that chronic stress exposure is accompanied by alteration of GR-regulated genes transcription mainly in the PFC and by modification that occur at epigenetic levels. Moreover, the ability of the pharmacological intervention with the antipsychotic LUR in normalizing the expression of selected genes in stressed rats is associated with a modification of their DNA methylation pattern. Furthermore, we found that the modifications that seem to occur in association with stress were still present following a period of washout from stress.

Despite the well-established involvement of the hippocampus in the regulation of the HPA axis activity, as well as in the modulation of stress response and the antidepressants activity, here we did not find any changes in either the ventral or in the dorsal hippocampal subregions of the GR-responsive genes considered.

Differently, we found that chronic mild stress decreased *Gadd45β* mRNA levels in PFC, indicating that the regulatory mechanisms involved in the transcription of this gene may be affected by stress exposure. This result is in line with the findings of Grassi and colleagues who demonstrated that chronic mild unpredictable stress produced a significant downregulation of the demethylase *Gadd45β* mRNA levels in the PFC of mice [38]. Interestingly, this transcriptional change was sustained by epigenetic modification, with an overall stress-induced increase of the methylation percentage at the GRE of Gadd45*β*. We hypothesize that chronic stress-induced methylation of these CGs could prevent the binding of transcriptional regulatory proteins at the GRE site, thus altering the downstream activity mediated by GR. Interestingly, the methylation status of Gadd45*β* was still elevated after three weeks washout from stress, suggesting an enduring effect of the adverse manipulation. In particular, we observed that specific cytosines are more methylated in stress conditions, indicating that further studies are needed to better clarify the role of each cytosine in controlling gene transcription.

Moreover, chronic lurasidone treatment was able to normalize not only the CMS-induced reduction of *Gadd45β* expression, but also the changes in the DNA methylation status due to chronic stress. These results suggest that lurasidone may be able to counteract the effects of stress, also by interfering with the epigenetic alterations produced by the adverse experience. The finding that chronic lurasidone treatment was able to prevent the DNA methylation changes when chronically administered during the pathological condition indeed may represent a new observation to deeper explore in the field of the “targeted interventions”, to alleviate adverse phenotype [39].

In line with the CMS-modulation of Gadd45β, prolonged stress also had persistent consequences on Sgk1 methylation in the CGs of the GRE, independently from lurasidone administration, even if the degree of changes was less marked than the one observed for the Gadd45β. Accordingly, Sgk1 has been identified as a DNA methylation biomarker of MDD since its methylation status is altered in the peripheral blood of depressed patients [40]. Moreover, stress also has negative effects on *Gilz* expression, both at the end of the stress procedure and following the rest period. These data are in line with the evidence that this gene, transcriptionally activated by glucocorticoids, is altered in the brain tissue of depressed patients [41,42]. However, we cannot link this modulation with the mechanism of DNA methylation at the GRE, since Gilz does not contain CGs in the proximity of its GRE, possibly suggesting that a different epigenetic mechanism drives GR-related transcription of this gene.

The reduction of the transcription of miR-143-3p seems to be in contrast with the decrease of *Gilz* expression due to stress exposure, suggesting that in our experimental conditions, changes in the activity of this miRNA mainly affect other target genes or may alter more the protein concentration than the mRNA levels.

The main limitation of this study is that we used two different protocols of stress, the CMS and the CRS. However, several studies (also provided by our group) demonstrated that both the paradigms lead to similar alterations both at behavioral [6,34,43] and molecular levels with impairment of neuroplastic mechanisms within the prefrontal cortex [33,43,44], suggesting that the neuronal circuitry involved in the stress response may, at least in part, overlap.

Moreover, seeing that glucocorticoids affect gene expression in non-neuronal-cell [45] additional studies, may clarify which cellular populations are mainly involved in the effects observed.

The results obtained in this work indicated that, among the genes considered in this study, chronic stress affected and compromised mainly *Gadd45β* expression in PFC, acting at both transcriptional and epigenetic levels. These effects were present even after the rest period, pointing out that *Gadd45β* may represent a stable, stress-induced molecular scar in the rat PFC. Moreover, the observation that both the expression and the methylation of this GR responsive gene in the proximity of the GRE site was completely prevented by the lurasidone treatment indicated that the modulation of Gadd45β may underlie the beneficial activity of lurasidone on the depressive-like behavior.

## 4. Materials and Methods

Adult male Wistar rats (Charles River, Germany, and Italy, respectively, for the first and second experiments) (post-natal day (pnd) 60) were brought into the laboratory one month before the start of the experiment. The animals were housed with food and water freely available and were maintained on a 12-h light/dark cycle and in constant temperature (22 ± 2 °C) and humidity (50 ± 5%) conditions.

Experiment 1: The CMS procedures used in this study have conformed to the rules and principles of the 86/609/EEC Directive and have been approved by the Local Bioethical Committee at the Maj Institute of Pharmacology Polish Academy of Sciences, Krakow, Poland.

Experiment 2: The procedures used for the CRS study have conformed to the rules and principles of the 2010/63/UE Directive, according to the authorizations from the Health Ministry n 151/2017-PR.

The animal studies are reported in compliance with the ARRIVE guidelines [46].

The molecular analyses were conducted in the PFC, dHip, and vHip. The PFC (defined as Cg1, Cg3, and IL subregions corresponding to plates 6–10 according to the atlas of Paxinos and Watson [47]) was dissected from 2-mm-thick slices whereas the dHip and vHip (plates 25–33 and plates 34–43, respectively, according to the atlas of Paxinos and Watson [47]) were dissected from the whole brain. Rats were killed 24 h after the last stressor or last pharmacological injection. The brain specimens were then rapidly frozen in dry ice and stored at −80 °C for molecular analyses.

### 4.1. Chronic Stress Procedure

Experiment 1: Chronic mild stress (CMS). After 3 weeks of adaptation to laboratory and housing conditions, the rats were randomly divided into two groups. One group (no stress) was housed in separate rooms and had no contact with the stressed animals while the other group of animals was subjected to the CMS procedure for 7 consecutive weeks. Briefly, animals were exposed to periods of 45 degree cage tilt, low-intensity stroboscopic illumination, soiled cage, paired housing, food or water deprivation, and intermittent illumination (see [6,33,34] for details).

Experiment 2: Chronic restraint stress (CRS). After two weeks of adaptation to laboratory and housing conditions, rats were divided into two groups (no stress and CRS). CRS group rats were exposed to unpredictable chronic restraint stress for 4 weeks. Rats were placed in an air-assessable cylinder for 1 h two times/day at random hours, to avoid habituation. The dimensions of the restrainers were similar to the size of the animal, which made the animal almost immobile in it. Rats (both unstressed and stressed) were then left undisturbed in their home cages for three weeks of washout.

### 4.2. Drug Treatment

Experiment 1: Both control and CMS groups, after two weeks of stress protocol, were each divided further into subgroups, and for subsequent five weeks, they received daily administration of vehicles (1% (*w*/*v*) hydroxyethylcellulose, 1 mL/kg, oral gavage) or LUR (3 mg/kg, oral gavage) (Sumitomo Dainippon Pharma Co. Ltd., Osaka, Japan). The drugs were administered at approximately 10.00 a.m. with a volume of 1 mL/kg

### 4.3. Sucrose Consumption Test and Body Weight

After a period of training to consume a 1% sucrose solution (sucrose was dissolved in tap water), following 14 h food and water deprivation, sucrose intake was measured by weighing pre-weighed bottles containing the sucrose solution at the end of the test. Subsequently, sucrose consumption was monitored, under similar conditions, at weekly intervals throughout the entire experiment. Body weight was monitored during the experiment and, in particular, the weight of the animals was recorded at the start of the experiment, after 3 weeks of CMS (that corresponded to the first week of LUR administration) and at the end of the stress procedure (corresponding to the seventh week of CMS and fifth week of LUR treatment).

### 4.4. Bioinformatic for miRNA/Integrated miRNA/mRNA Network Analysis

The identification of miRNAs to be measured was carried out through TargetScan [48], a tool useful to predict conserved sites for miRNA families broadly conserved among vertebrates. *Gadd45β*, *Sgk-1,* and *Gilz* mRNAs were scanned and only the conserved, but not the poorly conserved, miRNA families were considered.

The identified miRNA families were counterchecked with two different Diana Tools, MicroT-CDS [49,50] and miRDB [51,52], and two online databases for miRNA target prediction and functional annotations, using them as probes for fishing out target mRNAs.

### 4.5. RNA Preparation and Real Time RT-PCR

Total RNA samples, including miRNAs, were extracted using the AllPrep DNA/RNA/miRNA Universal Kit (Qiagen, Milano, Italy), according to the manufacturer’s instructions, and quantified by spectrophotometric analysis (see [53] for details). The samples were then processed for the real-time polymerase chain reaction (PCR) to assess mRNA levels of *Gadd45β*, *Sgk1*, and *Gilz* (Table S3a,b).

The mRNA levels were analyzed by the TaqMan qRT–PCR instrument (CFX384 real-time system, Bio-Rad Laboratories S.r.l., Segrate, Italy) using the iScript one-step RT–PCR kit for probes (Bio-Rad Laboratories S.r.l., Segrate, Italy) as previously reported [6]. Samples (10 ng/uL) were run in 384-well format in triplicates as multiplexed reactions with a normalizing internal control (36B4). A comparative cycle threshold (Ct) method was used to calculate the relative target gene expression with the 2^−∆(∆CT)^ method [54].

The expression levels of miR-452-3p, miR-19a-3p, miR-19b-3p, and miR-143-3p were analyzed by the RT-PCR using the Taqman MicroRNA (Thermofisher, Monza, Italy) (Table S3b). assays and the CFX384 instrument. The relative expression of miRNAs was normalized to the levels of the U6.

### 4.6. DNA Extraction, Bisulfite Treatment, and DNA Methylation Analyses

DNA samples were extracted using the AllPrep DNA/RNA/miRNA Universal Kit (Qiagen, Milano, Italy) and stored at −80 °C. An aliquot of 0.5 μg of DNA was treated with sodium-bisulfite with the EZ-96 DNA Methylation-Gold kit (Zymo Research, Irvine, CA, USA) and immediately further processed. Both procedures were executed according to the manufacturer’s instructions. DNA methylation analysis was carried out using bisulfite-PCR-pyrosequencing. The bisulfite-treated genomic DNA samples were amplified with PCR for the regions of interest. CpG sites within the glucocorticoid responsive element of Gadd45β and Sgk1 were investigated (Figure 2). Detailed information concerning primer sequences and genomic regions is listed in Table S4. Pyrosequencing of the PCR products was carried out using the PyroMark MD Pyrosequencing System (Qiagen, Milano, Italy) [55]. The percentage of 5-methylcytosine (% 5mC) was presented as the percentage of cytosine that was methylated divided by the sum of methylated and unmethylated cytosines.

### 4.7. Data and Statistical Analysis

All the data were analyzed by using the “IBM SPSS Statistics”, version 26. The whole dataset presented passed the normality test in the Kolmogorov–Smirnov test and the Shapiro–Wilk tests as well as the Spearman’s test for homoscedasticity. The effect of chronic mild stress and of the pharmacological treatment, as independent factors, were analyzed with the two-way ANOVA, and were followed, when appropriate, by Tukey multiple comparison’s test). Each experimental group consisted of 8–10 rats. The results obtained in stressed rats after the period of washout were analyzed by the Unpaired- t test. Benjamini–Hochberg multiple testing correction for false discovery rate (FDR) was applied and significance set to FDR adjusted *p*-value  <  0.05.

Significance for all tests was assumed for *p* < 0.05. Data are presented as mean of no-stress rats ± standard error (SEM). Each experimental group consisted of 9–10 animals.

## 5. Conclusions

In conclusion, our data highlight that chronic stress exposure results in persistent changes in DNA methylation in specific regions of genes related to glucocorticoids signaling and that lurasidone may acts as a modifier of such mechanisms, suggesting its potential as a modulator of the HPA axis, that is compromised in different psychiatric pathologies [56]. Moreover, these epigenetic alterations may be connected with the behavioral deficits we observed in animal models based on chronic stress exposure useful for the study of depression [33,34,44]. Further, it may be inferred that the rescue of these symptoms by lurasidone treatment [6] may be linked with the possible properties of the drug as “epigenetic modulators”, although other studies are needed to more deeply elucidate this potential effect of lurasidone.

## Figures and Tables

**Table 1:** (**a**,**b**): Analysis of miRNAs levels in chronically stressed rats treated with lurasidone (**a**) and after a period of washout from chronic stress exposure (**b**). The data, expressed as a percentage of no stress/VEH animals (set at 100%), are the mean ± SEM.U6 was used as internal controls. * *p* < 0.05 vs. No stress/VEH-No stress, ## *p* < 0.05 vs. CMS/VEH (Two-way ANOVA with Tukey multiple comparison’s test).

**(a)**	**No Stress**		**CMS**	
	**Vehicle**	**Lurasidone**	**Vehicle**	**Lurasidone**
miR-452-3p (U6)	100 ± 20	74 ± 14	91 ± 25	86 ± 18
miR-19a-3p (U6)	100 ± 12	178 ± 24 *	116 ± 15	231 ± 32 ##
miR-19b-3p (U6)	100 ± 16	85 ± 13	114 ± 25	115 ± 21
miR-143-3p (U6)	100 ± 13	104 ± 17	61 ± 11	95 ± 13
**(b)**	**No Stress**	**CRS + Washout**		
miR-452-3p (U6)	100 ± 26	62 ± 15		
miR-19a-3p (U6)	100 ± 21	105 ± 25		
miR-19b-3p (U6)	100 ± 20	80 ± 16		
miR-143-3p (U6)	100 ± 13	63 ± 10 *		

## Supplementary Materials

The following are available online at https://www.mdpi.com/article/10.3390/ijms22126197/s1.

## References

[1] Caspi A. (2003). Influence of Life Stress on Depression: Moderation by a Polymorphism in the 5-HTT Gene. Science.

[2] Fava M., Kendler K.S. (2000). Major Depressive Disorder. Neuron.

[3] McEwen B.S., Bowles N.P., Gray J.D., Hill M.N., Hunter R.G., Karatsoreos I.N., Nasca C. (2015). Mechanisms of stress in the brain. Nat. Neurosci..

[4] Holsboer F. (2000). The Corticosteroid Receptor Hypothesis of Depression. Neuropsychopharmacology.

[5] McEwen B.S. (2006). Protective and damaging effects of stress mediators: Central role of the brain. Dialogues Clin. Neurosci..

[6] Calabrese F., Brivio P., Sbrini G., Gruca P., Lason M., Litwa E., Niemczyk M., Papp M., Riva M.A. (2020). Effect of lurasidone treatment on chronic mild stress-induced behavioural deficits in male rats: The potential role for glucocorticoid receptor signalling. J. Psychopharmacol..

[7] Krishnan V., Nestler E.J. (2010). Linking Molecules to Mood: New Insight into the Biology of Depression. Am. J. Psychiatry.

[8] Frieling H., Tadić A. (2013). Value of genetic and epigenetic testing as biomarkers of response to antidepressant treatment. Int. Rev. Psychiatry.

[9] Szyf M., McGowan P.O., Turecki G., Meaney M.J., Worthman C.M., Plotsky P.M., Schechter D.S., Cummings C.A. (2010). The Social Environment and the Epigenome. Formative Experiences.

[10] Dwivedi Y. (2011). Evidence demonstrating role of microRNAs in the etiopathology of major depression. J. Chem. Neuroanat..

[11] Baudry A., Mouillet-Richard S., Schneider B., Launay J.-M., Kellermann O. (2010). MiR-16 Targets the Serotonin Transporter: A New Facet for Adaptive Responses to Antidepressants. Science.

[12] Farrell C., O’Keane V. (2016). Epigenetics and the glucocorticoid receptor: A review of the implications in depression. Psychiatry Res..

[13] Auger C.J., Auger A.P. (2013). Permanent and plastic epigenesis in neuroendocrine systems. Front. Neuroendocrinol..

[14] Zovkic L.B., Meadows J.P., Kaas G.A., Sweatt J.D. (2013). Interindividual variability in stress susceptibility: A role for epigenetic mechanisms in PTSD. Front. Psychiatry.

[15] Krishnan V., Nestler E.J. (2008). The molecular neurobiology of depression. Nature.

[16] Uchida S., Yamagata H., Seki T., Watanabe Y. (2018). Epigenetic mechanisms of major depression: Targeting neuronal plasticity. Psychiatry Clin. Neurosci..

[17] Zannas A.S., Chrousos G.P. (2017). Epigenetic programming by stress and glucocorticoids along the human lifespan. Mol. Psychiatry.

[18] Vinet L., Zhedanov A. (2010). A “missing” family of classical orthogonal polynomials. Neuropsychopharmacology.

[19] Weaver I.C.G., Cervoni N., Champagne F.A., D’Alessio A.C., Sharma S., Seckl J.R., Dymov S., Szyf M., Meaney M.J. (2004). Epigenetic programming by maternal behavior. Nat. Neurosci..

[20] Murgatroyd C., Patchev A.V., Wu Y., Micale V., Bockmühl Y., Fischer D., Holsboer F., Wotjak C.T., Almeida O.F.X., Spengler D. (2009). Dynamic DNA methylation programs persistent adverse effects of early-life stress. Nat. Neurosci..

[21] Shimizu S., Tanaka T., Takeda T., Tohyama M., Miyata S. (2015). The Kampo Medicine Yokukansan Decreases MicroRNA-18 Expression and Recovers Glucocorticoid Receptors Protein Expression in the Hypothalamus of Stressed Mice. Biomed Res. Int..

[22] Vreugdenhil E., Verissimo C.S.L., Mariman R., Kamphorst J.T., Barbosa J.S., Zweers T., Champagne D.L., Schouten T., Meijer O.C., Ron de Kloet E. (2009). MicroRNA 18 and 124a Down-Regulate the Glucocorticoid Receptor: Implications for Glucocorticoid Responsiveness in the Brain. Endocrinology.

[23] Li M., D’Arcy C., Li X., Zhang T., Joober R., Meng X. (2019). What do DNA methylation studies tell us about depression? A systematic review. Transl. Psychiatry.

[24] Story Jovanova O., Nedeljkovic I., Spieler D., Walker R.M., Liu C., Luciano M., Bressler J., Brody J., Drake A.J., Evans K.L. (2018). DNA Methylation Signatures of Depressive Symptoms in Middle-aged and Elderly Persons. JAMA Psychiatry.

[25] Farrell C., Doolin K., O’ Leary N., Jairaj C., Roddy D., Tozzi L., Morris D., Harkin A., Frodl T., Nemoda Z. (2018). DNA methylation differences at the glucocorticoid receptor gene in depression are related to functional alterations in hypothalamic–pituitary–adrenal axis activity and to early life emotional abuse. Psychiatry Res..

[26] Anacker C., Cattaneo A., Musaelyan K., Zunszain P.A., Horowitz M., Molteni R., Luoni A., Calabrese F., Tansey K., Gennarelli M. (2013). Role for the kinase SGK1 in stress, depression, and glucocorticoid effects on hippocampal neurogenesis. Proc. Natl. Acad. Sci. USA.

[27] Gavin D.P., Sharma R.P., Chase K.A., Matrisciano F., Dong E., Guidotti A. (2012). Growth Arrest and DNA-Damage-Inducible, Beta (GADD45b)-Mediated DNA Demethylation in Major Psychosis. Neuropsychopharmacology.

[28] Ayroldi E., Riccardi C. (2009). Glucocorticoid-induced leucine zipper (GILZ): A new important mediator of glucocorticoid action. FASEB J..

[29] Itani O.A., Liu K.Z., Cornish K.L., Campbell J.R., Thomas C.P. (2002). Glucocorticoids stimulate human sgk1 gene expression by activation of a GRE in its 5′-flanking region. Am. J. Physiol. Endocrinol. Metab..

[30] Muzikar K.A., Nickols N.G., Dervan P.B. (2009). Repression of DNA-binding dependent glucocorticoid receptor-mediated gene expression. Proc. Natl. Acad. Sci..

[31] Anacker C., Zunszain P.A., Cattaneo A., Carvalho L.A., Garabedian M.J., Thuret S., Price J., Pariante C.M. (2011). Antidepressants increase human hippocampal neurogenesis by activating the glucocorticoid receptor. Mol. Psychiatry.

[32] Asselin-Labat M.-L., David M., Biola-Vidamment A., Lecoeuche D., Zennaro M.-C., Bertoglio J., Pallardy M. (2004). GILZ, a new target for the transcription factor FoxO3, protects T lymphocytes from interleukin-2 withdrawal–induced apoptosis. Blood.

[33] Calabrese F., Savino E., Papp M., Molteni R., Riva M.A. (2016). Chronic mild stress-induced alterations of clock gene expression in rat prefrontal cortex: Modulatory effects of prolonged lurasidone treatment. Pharmacol. Res..

[34] Calabrese F., Brivio P., Gruca P., Lason-Tyburkiewicz M., Papp M., Riva M.A. (2017). Chronic Mild Stress-Induced Alterations of Local Protein Synthesis: A Role for Cognitive Impairment. ACS Chem. Neurosci..

[35] Caputo V., Ciolfi A., Macri S., Pizzuti A. (2015). The Emerging Role of MicroRNA in Schizophrenia. CNS Neurol. Disord. Drug Targets.

[36] Kocerha J., Dwivedi Y., Brennand K.J. (2015). Noncoding RNAs and neurobehavioral mechanisms in psychiatric disease. Mol. Psychiatry.

[37] Luoni A., Riva M.A. (2016). MicroRNAs and psychiatric disorders: From aetiology to treatment. Pharmacol. Ther..

[38] Grassi D., Franz H., Vezzali R., Bovio P., Heidrich S., Dehghanian F., Lagunas N., Belzung C., Krieglstein K., Vogel T. (2017). Neuronal Activity, TGFβ-Signaling and Unpredictable Chronic Stress Modulate Transcription of Gadd45 Family Members and DNA Methylation in the Hippocampus. Cereb. Cortex.

[39] Szyf M., Tang Y.-Y., Hill K.G., Musci R. (2016). The dynamic epigenome and its implications for behavioral interventions: A role for epigenetics to inform disorder prevention and health promotion. Transl. Behav. Med..

[40] Numata S., Ishii K., Tajima A., Iga J., Kinoshita M., Watanabe S., Umehara H., Fuchikami M., Okada S., Boku S. (2015). Blood diagnostic biomarkers for major depressive disorder using multiplex DNA methylation profiles: Discovery and validation. Epigenetics.

[41] Pandey G.N., Rizavi H.S., Ren X., Dwivedi Y., Palkovits M. (2013). Region-specific alterations in glucocorticoid receptor expression in the postmortem brain of teenage suicide victims. Psychoneuroendocrinology.

[42] Frodl T., Carballedo A., Hughes M.M., Saleh K., Fagan A., Skokauskas N., McLoughlin D.M., Meaney J., O’Keane V., Connor T.J. (2012). Reduced expression of glucocorticoid-inducible genes GILZ and SGK-1: High IL-6 levels are associated with reduced hippocampal volumes in major depressive disorder. Transl. Psychiatry.

[43] Brivio P., Sbrini G., Corsini G., Paladini M.S., Racagni G., Molteni R., Calabrese F. (2020). Chronic Restraint Stress Inhibits the Response to a Second Hit in Adult Male Rats: A Role for BDNF Signaling. Int. J. Mol. Sci..

[44] Luoni A., Macchi F., Papp M., Molteni R., Riva M.A. (2015). Lurasidone Exerts Antidepressant Properties in the Chronic Mild Stress Model through the Regulation of Synaptic and Neuroplastic Mechanisms in the Rat Prefrontal Cortex. Int. J. Neuropsychopharmacol..

[45] Piechota M., Korostynski M., Golda S., Ficek J., Jantas D., Barbara Z., Przewlocki R. (2017). Transcriptional signatures of steroid hormones in the striatal neurons and astrocytes. BMC Neurosci..

[46] McGrath J.C., Lilley E. (2015). Implementing guidelines on reporting research using animals (ARRIVE etc.): New requirements for publication in BJP. Br. J. Pharmacol..

[47] Paxinos G., Watson C. (2004). The Rat Brain in Stereotaxic Coordinates the New Coronal Set.

[48] Agarwal V., Bell G.W., Nam J.-W., Bartel D.P. (2015). Predicting effective microRNA target sites in mammalian mRNAs. eLife.

[49] Paraskevopoulou M.D., Georgakilas G., Kostoulas N., Vlachos I.S., Vergoulis T., Reczko M., Filippidis C., Dalamagas T., Hatzigeorgiou A.G. (2013). DIANA-microT web server v5.0: Service integration into miRNA functional analysis workflows. Nucleic Acids Res..

[50] Reczko M., Maragkakis M., Alexiou P., Grosse I., Hatzigeorgiou A.G. (2012). Functional microRNA targets in protein coding sequences. Bioinformatics.

[51] Chen Y., Wang X. (2020). miRDB: An online database for prediction of functional microRNA targets. Nucleic Acids Res..

[52] Liu W., Wang X. (2019). Prediction of functional microRNA targets by integrative modeling of microRNA binding and target expression data. Genome Biol..

[53] Brivio P., Sbrini G., Riva M.A., Calabrese F. (2020). Acute Stress Induces Cognitive Improvement in the Novel Object Recognition Task by Transiently Modulating Bdnf in the Prefrontal Cortex of Male Rats. Cell. Mol. Neurobiol..

[54] Livak K.J., Schmittgen T.D. (2001). Analysis of Relative Gene Expression Data Using Real-Time Quantitative PCR and the 2−ΔΔCT Method. Methods.

[55] Bollati V., Baccarelli A., Hou L., Bonzini M., Fustinoni S., Cavallo D., Byun H.-M., Jiang J., Marinelli B., Pesatori A.C. (2007). Changes in DNA Methylation Patterns in Subjects Exposed to Low-Dose Benzene. Cancer Res..

[56] Pariante C.M., Lightman S.L. (2008). The HPA axis in major depression: Classical theories and new developments. Trends Neurosci..

