# Supplementary material for: Stress Modifies the Expression of Glucocorticoid-Responsive Genes by Acting at Epigenetic Levels in the Rat Prefrontal Cortex: Modulatory Activity of Lurasidone
Source: Int J Mol Sci. 2021 Jun 8;22(12):6197. doi: 10.3390/ijms22126197 (PMC8228132; doi:10.3390/ijms22126197)
Supplement: Supplementary file 1 [file ijms-22-06197-s001.zip › ijms-1204248-supplementary.pdf]

<u>Supplementary materials</u>

a) Body weight (g)	No stress		CMS	
	Vehicle	Lurasidone	Vehicle	Lurasidone
Baseline	338±7	332±8	336±8	342±8
Week 3	358±8	356±9	343±9	349±9
Week 7	391±9	391±8,	363±10 *	374 ±11
b) Sucrose intake (g)				
Baseline	14,9±1,3	13,8±1,1	12,6±0,5	12,6±0,4
Week 3	13,8±1,9	11,3±1,2	6,9±0,9 **	7,0±0,7
Week 7	14,4±1,6	12,4±1,4	6,9±0,5 **	12,0±1,1 #
c) Sucrose intake/body weight				
Baseline	0,044±0,004	0,042±0,003	0,038±0,002	0,037±0,002
Week 3	0,039±0,006	0,032±0,004	0,021±0,003 **	0,020±0,002
Week 7	0,037±0,004	0,032±0,004	0,019±0,002 **	0,032±0,003 #

Table S1. Effects of chronic mild stress and of lurasidone treatment on body weight and of the sucrose intake measures at the start of the CMS procedure (baseline) and after 3 and 7 weeks of CMS. The data, expressed as grams of body weight or sucrose intake, are the mean ± SEM. *$p<0.05$; **$p<0.01$ vs No stress/VEH; # $p<0.05$ vs CMS/VEH (Two-way ANOVA with Tukey multiple comparison's test).

a) vHip	No stress		CMS	
	Vehicle	Lurasidone	Vehicle	Lurasidone
Gadd45 β	100±9	92±8	97±7	96±11
Sgk1	100±5	87±4	106±6	77±5 ##
Gilz	100±8	102±4	91±6	92±5
**b) dHip**	**No stress**		**CMS**	
	Vehicle	Lurasidone	Vehicle	Lurasidone
Gilz	100±7	110±7	95±7	108±9

Table S2. Analysis of *Gadd45β*, *Sgk1* and *Gilz* mRNA levels in the ventral (a) and dorsal hippocampus (b) of chronically stressed rats: modulation by chronic lurasidone (LUR) treatment. The data, expressed as a percentage of no stress/VEH animals (set at 100%), are the mean ± SEM. ## $p<0.01$ vs CMS/VEH (Two-way ANOVA with Tukey multiple comparison's test).

a) Gene	Forward primer	Reverse primer	Probe
Sgk1	GGTGGGTGGCTCTGAAGAAT	ACTCCACCCAGTTCTTCACC	GATCCAGAACCACATGAATGGG
Gilz	CGGTCTATCAACTGCACAATTTC	CTTCACTAGATCCATGGCCTG	AACGGAAACCACATCCCCTCCAA
36b4	TCAGTGCCTCACTCCATCAT	AGGAAGGCCTTGACCTTTTC	TGGATACAAAAGGGTCCTGG

b) Gene	Accession number	Assay ID
Gadd45β	BC085337.1	Rn01452530_gl
miR-452-3p	MIMAT0035749	
miR-19a-3p	MIMAT0000789	
miR-19b-3p	MIMAT0000788	
miR-143-3p	MIMAT0000849	
U6	NR_004394	

Table S3. a) Sequences of forward and reverse primers and probes used in Real-time PCR analyses and purchased from EurofinsMWG-Operon. b) Probes purchased from Life Technologies, which did not disclose the sequences.

Gene	Chromosome position (RGSC_6.0)	Region	CpG loci	Primers (F), reverse (R) and sequencing (S)
Gadd45 *β*	Chr7:11,646,283-11,648,338	exon	5	F: Biotin-GTTAAGATAGGAAGGAGGGGATTT<br>R: AAAACAAGAAAACTTAACCAATTT<br>S: ACAATTCACTATCCAAC
Sgk1	Chr1:24,185,451-24,302,309	promotor	2	F: GTATTAGGGTAAGGGTATTGATT<br>R: Biotin-TCATTTCACTTTTTTTTCCAACTA<br>S: TTGTAAGGTTTAAAATTTAT

Table S4. Pyrosequencing assay information (F: forward; R: reverse; S: sequencing).
